# Mechanistic analysis of actin-binding compounds that affect the kinetics of cardiac myosin–actin interaction

**DOI:** 10.1016/j.jbc.2021.100471

**Published:** 2021-02-25

**Authors:** Osha Roopnarine, David D. Thomas

**Affiliations:** Department of Biochemistry, Molecular Biology and Biophysics, University of Minnesota, Minneapolis, Minnesota , USA

**Keywords:** actin, drug action, compound, myosin, fluorescence, kinetics, ATPase, mechanism, inhibition, DCM, dilated cardiomyopathy, ELC, essential light chain, FRET, fluorescence resonance energy transfer, HCM, hypertrophic cardiomyopathy, HTS, high-throughput screen, Mava, mavacamten, OM, omecamtiv mecarbil, PFA, pyrene-labeled F-actin, PIA, pyrene iodoacetamide

## Abstract

Actin–myosin mediated contractile forces are crucial for many cellular functions, including cell motility, cytokinesis, and muscle contraction. We determined the effects of ten actin-binding compounds on the interaction of cardiac myosin subfragment 1 (S1) with pyrene-labeled F-actin (PFA). These compounds, previously identified from a small-molecule high-throughput screen (HTS), perturb the structural dynamics of actin and the steady-state actin-activated myosin ATPase activity. However, the mechanisms underpinning these perturbations remain unclear. Here we further characterize them by measuring their effects on PFA fluorescence, which is decreased specifically by the strong binding of myosin to actin. We measured these effects under equilibrium and steady-state conditions, and under transient conditions, in stopped-flow experiments following addition of ATP to S1-bound PFA. We observed that these compounds affect early steps of the myosin ATPase cycle to different extents. They increased the association equilibrium constant *K*_1_ for the formation of the strongly bound collision complex, indicating increased ATP affinity for actin-bound myosin, and decreased the rate constant *k*_*+*2_ for subsequent isomerization to the weakly bound ternary complex, thus slowing the strong-to-weak transition that actin–myosin interaction undergoes early in the ATPase cycle. The compounds' effects on actin structure allosterically inhibit the kinetics of the actin–myosin interaction in ways that may be desirable for treatment of hypercontractile forms of cardiomyopathy. This work helps to elucidate the mechanisms of action for these compounds, several of which are currently used therapeutically, and sets the stage for future HTS campaigns that aim to discover new drugs for treatment of heart failure.

Actin is abundant in eukaryotic cells and is involved in many important cellular processes such as movement, cell division, maintenance of cellular shape, transport of vesicles, phagocytosis, and contractility (1). Actin interacts with and is regulated by many actin-binding proteins, such as capping and severing proteins, nucleating proteins, and stabilizers. Actin interacts with myosin to generate force and movement, which is regulated in the muscle by proteins such as tropomyosin, troponin, and myosin-binding protein C.

In striated muscle contraction, actin and myosin interactions are nucleotide-dependent, as the hydrolysis of ATP by myosin provides energy for the structural and affinity changes that result in force. In rigor, actin (A) and myosin (M) bind strongly (S) in a stereospecific manner in the absence of ATP to form the actin–myosin complex (A.M), in which the myosin head (catalytic domain, CD, and light-chain domain, LCD, also called lever arm) is straight (Fig. 1). This changes upon ATP (T) binding to the myosin active site (A.M.T), which induces weak binding (W) in the strong-to-weak (S→W) transition, resulting in dissociation of myosin from actin (M.T) (Fig. 2). Subsequent structural changes in myosin reorient the LCD to produce a bent myosin head and ATP hydrolysis (producing M.D.P_i_) primes myosin for weak-binding (W) reattachment to actin (A.M.D.P_i_) (2). This induces the reorientation of the LCD (bent-to-straight) to produce the force-generating powerstroke and subsequent release of P_i_ to produce the strongly bound (S) A.M.ADP state (3). The release of ADP to form the A.M complex completes the actin–myosin ATPase cycle (4, 5). Thus, the key actin–myosin interaction during force production, involving the weak-to-strong transition (W→S), can be fine-tuned by changes in either LCD structure (flexibility or axial rotations) or equilibrium/rate constants leading to this transition (6, 7, 8).

**Figure 1:**
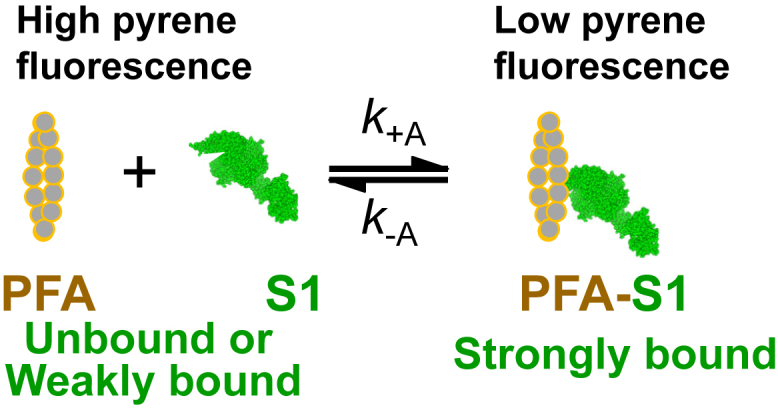
**Actin (PFA) binding to and dissociation from myosin S1.** The equilibrium constant for dissociation, *K*_A_, was determined from the ratio of the rate constants *k*_−A_/*k*_+A_ (Equation 9).

**Figure 2:**
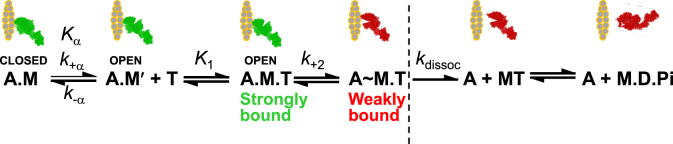
**ATP (T)-induced dissociation of the actin (A)–myosin (M) complex.** The strongly bound conformation of the myosin head is shown in green and the weakly bound or detached conformation is shown in red. *K*_α_ is the association equilibrium constant for the isomerization of actin–myosin from the closed (A.M) to open (A.M′) conformation of the nucleotide-binding site. *K*_1_ (Equation 10) is the association equilibrium constant for ATP binding to myosin that is strongly bound to actin (sometimes referred to as *K*_T_), *k*_+2_ (Equation 11) is the forward rate constant for isomerization of the collision complex (A.M.T) to the weakly bound ternary complex (A∼M.T), and *k*_dissoc_ is the rate constant for dissociation of that complex. This paper focuses on the effects of compounds on *K*_1_ and *k*_+2_ described before the dashed line in the scheme.

Actin has clinical relevance, as both skeletal and cardiac actin isoforms have mutations that cause human diseases (9). Skeletal muscle actin mutations account for about 20% of congenital myopathies and occur as nemaline, intranuclear rod and/or actin myopathy, and congenital fiber-type disproportion (9, 10). Even more importantly, cardiac actin mutations that appear in the myosin or tropomyosin-binding regions cause either hypertrophic (HCM) or dilated (DCM) cardiomyopathies (11), increasing the motivation to discover small molecules that target actin for therapeutic treatment of actin-related diseases (12, 13).

Small molecules have recently been investigated as potential therapeutic agents for myosin mutations that cause inherited heart disease, with particular focus on hypertrophic cardiomyopathy (HCM). These potential drugs include omecamtiv mecarbil (OM) (14, 15) and mavacamten (Mava), formerly known as MYK461 (16), both of which were developed from HTS screens targeting myosin. It was shown that OM increases contractility of the cardiac muscle and is an activator of actin-activated myosin ATPase (2, 13, 14, 15). More specifically, OM increases the equilibrium constant for ATP hydrolysis by myosin (17), increases phosphate release (14), and slows the powerstroke or LCD rotation (2). Also, structural studies show that OM binds to stabilize myosin in a pre-powerstroke state in which the LCD is primed to transition into the powerstroke, which results in an increase in the number of myosins that are actin-attached in a force-producing state (18) and thus, making OM a potential activator candidate for therapeutics of heart failure. Mava inhibits the actin-activated rates of myosin ATPase activity and phosphate release (12, 16, 19, 20) and is hypothesized to stabilize the autoinhibited state of two-headed myosin that represents the interacting heads motif (IHM), the structural basis of the super-relaxed state of myosin in muscle fibers (6, 20, 21). Mava is in the third phase of clinical trials as a therapeutic for hypercontractility in HCM caused by myosin mutations (22). Recently, Mava was also proposed for treatment of HCM caused by mutations in the calcium regulatory thin filament protein troponin (23). However, the results of that study were mixed, suggesting that for those troponin-based HCM mutations, Mava would not be a suitable therapeutic candidate.

Small-molecule compounds designed for use as drugs to manage specific human diseases may affect other systems in the body, including the cardiovascular system. Therefore, it is important to determine the effects of these compounds on the contractility of cardiac and skeletal muscle. Because actin's role in muscle contractility (both striated and smooth) is regulated by many other proteins such as those in the thin filament (*e.g.*, tropomyosin, troponin, and caldesmon) or the thick filament (*e.g.*, myosin heavy chain, myosin light chains, and myosin binding protein C), it is also important to determine the effects of the actin-binding compounds on the functions of these associated proteins. The focus of the present study is to determine how previously identified actin-binding compounds affect the kinetics of the actin–myosin interaction.

A recently engineered biosensor consisting of donor-labeled actin and an acceptor-labeled peptide derived from the N terminus of the myosin essential light chain (ELC) was used in a fluorescence resonance energy transfer (FRET) high-throughput screen (HTS) of the NIH Clinical Collection (NCC), a library of compounds with a history of use and safety in human clinical trials (24, 25). In that study, ten “hit” compounds (Fig. 3) were found to affect actin–myosin function. These ten compounds were FDA-approved drugs for therapeutic treatment in diverse human diseases (Fig. 3). Car is used as a β-blocker for treatment of hypertension, Dan is used as a skeletal muscle relaxant, and Mef is used as an antimalarial. Thi, Flu, and Phn are used as antipsychotics. Flp, Hon, and Mit are used to treat cancer. Teg has been used to treat irritable bowel syndrome and constipation. However, Thi and Flp are no longer in common use because they cause liver toxicity. Dan also causes hepatoxicity at varying dosage and is under FDA caution for usage (26). Mit is also under FDA caution as it may contribute to heart failure, and Teg is no longer in use because it can contribute to heart failure. All ten of these compounds significantly decreased the FRET efficiency in the above-described HTS, indicating decreased myosin–actin interaction. Some of the compounds also had significant effects on actin filament flexibility and on steady-state actin-activated myosin S1 ATPase activities (24, 25).

**Figure 3:**
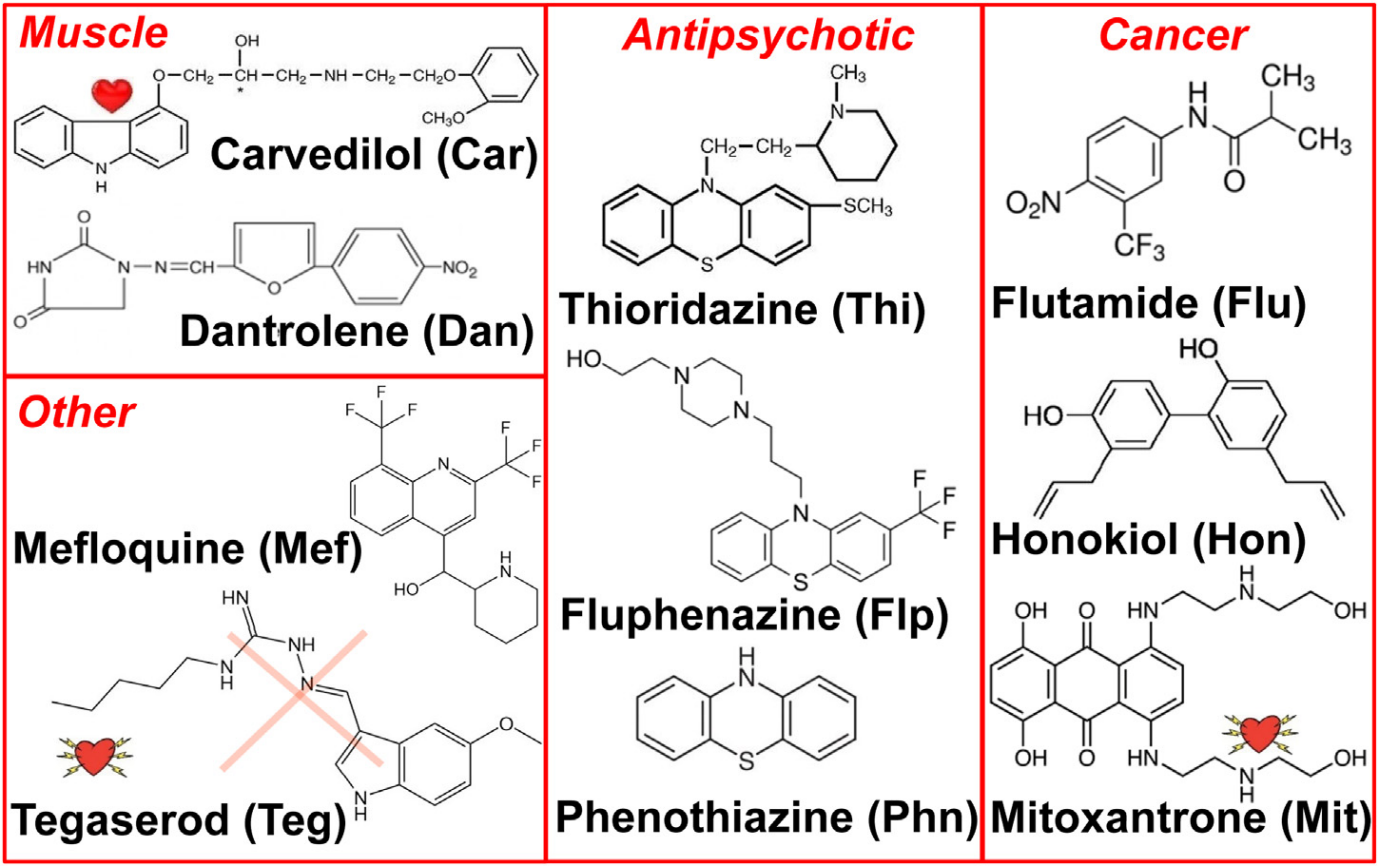
**Chemical structures for ten actin-binding compounds discovered from an HTS FRET assay.** The drugs are categorized by their intended application for treatment of human disease (see text for usage and warnings). The drugs will be referred to below by the indicated abbreviations.

A subsequent study by the same authors showed that some of these actin-binding compounds have distinct effects on the polymerization properties of skeletal and cardiac actin isoforms and on the steady-state ATPase activities of skeletal and cardiac myofibrillar ATPase activity (27). Given the very small sequence differences in structure between cardiac and skeletal muscle actin, this is a surprising finding. This is important because it provided motivation to further investigate the transient kinetics of the actin–myosin interaction, as steady-state kinetics does not provide direct information about fundamental rate and equilibrium constants, which are important for determining therapeutic relevance and providing insights into muscle mechanisms in health and disease.

In the present study, we investigate in more detail the mechanisms of action for these actin-binding compounds, focusing on cardiac muscle because of its high-potential therapeutic significance. Specifically, we ask the following question: Which steps early in the actin–myosin ATPase reaction do these compounds affect? A key tool in answering this question is the fluorescence of pyrene-labeled actin, which has been well characterized for detecting the kinetics of strong-binding myosin heads on actin (28).

Pyrene iodoacetamide (PIA) covalently binds to cysteine 374 (C374) in F-actin, which is proximal to, but not within, the myosin interaction sites on actin. C374 does not appear to be directly involved in the myosin–actin interaction (29), but it might be important for monomer-to-monomer contact in the actin filament, because deletion of C374 destabilizes F-actin (30). Labeling of C374 with PIA, producing pyrene-labeled F-actin (PFA), has no significant effects on the equilibrium or dynamics of myosin's interaction with actin, but when S1 binds to PFA in a strong-binding state, the fluorescence is decreased (quenched) because a local conformational change causes the pyrene moiety to become more accessible to the solution (31, 32). The decrease of PFA fluorescence in the presence of a compound indicates that the compound induces a conformational change that increases solvent exposure to the actin-bound pyrene, due to a decreased fraction of actin monomers occupied by strongly bound myosin.

In the present study, we first measured the impact of each compound on PFA fluorescence under equilibrium and steady-state conditions, in the absence and presence of bovine cardiac ventricular myosin S1 (β-S1) and ATP. We then used stopped-flow fluorescence to study the transient-state kinetics of the formation of the PFA-S1 complex and the effects of ATP binding to the myosin active site in the presence and absence of the compounds. Specifically, we determined how the compounds affect the binding affinity of actin and S1 in rigor (Fig. 1), the ATP affinity for myosin in the myosin–actin complex, and the subsequent rate of isomerization of the collision complex (A.M.T) to the ternary complex (A∼M.T) (Fig. 2), all of which were monitored by PFA fluorescence. We found that several of these compounds weaken the actin–myosin interaction in rigor, increase the ATP affinity for myosin, and decrease the rate constant of the subsequent isomerization step in the early phase of the ATPase cycle. These results support the hypothesis that actin-binding compounds allosterically affect the actin–myosin interaction by perturbing kinetic constants early in the myosin ATPase cycle.

## Results

### Characterization of pyrene-labeled actin

The ratio of bound pyrene to F-actin was 0.95–1.1, consistent with complete and specific reaction at Cys374 (28). Bovine ventricular myosin S1 (0.1 μM) had Mg-ATPase activity of 0.009 ± 0.04 s^−1^ in the absence of actin and 1.15 ± 0.03 s^−1^ in the presence of 15 μM phalloidin-stabilized F-actin, consistent with previous results (20, 33).

### Steady-state: pyrene-actin detects the strongly bound actin–myosin interaction

**Figure 4:**
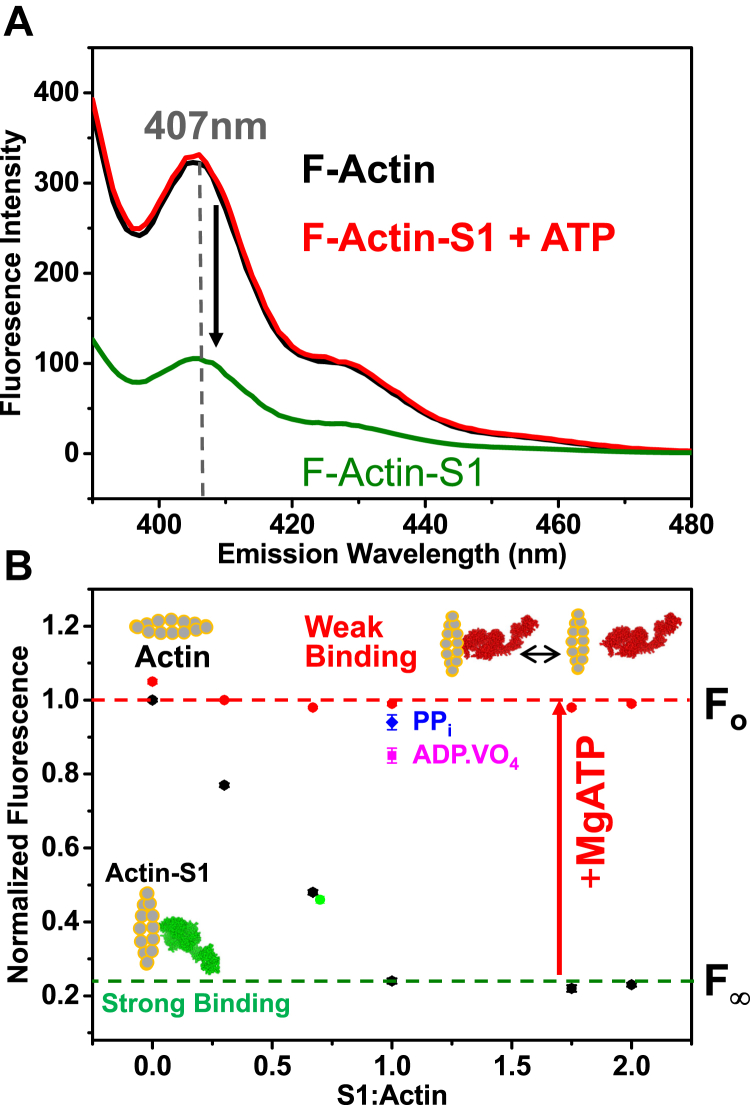
**Effects of S1 binding to pyrene-labeled F-actin (PFA) in the absence and presence of nucleotides.***A,* fluorescence spectra of 2 μM PFA (*black*) as a function of [S1] in the absence (*green*) and presence (*red*) of 1 mM Mg.ATP. *B*, inhibition of PFA fluorescence (at 407 nm) as a function of [S1] () and restoration by 1 mM MgATP (•). We also show the effects of forming the Mg.ADP.VO_4_ () and Mg.PP_i_ () complexes in equimolar PFA-S1 (2 μM). The PFA fluorescence of S1:Actin (0.7:1 M ratio) is shown as a green data point ().

As shown previously (28, 31), phalloidin-stabilized PFA has high fluorescence intensity with a maximum at 407 nm (Fig. 4*A*, black spectrum), and the addition of myosin S1 to pyrene-labeled F-actin linearly decreases the fluorescence intensity (Fig. 4*A*, green spectrum) until the molar ratio of S1 to Actin is 1, and then it becomes independent of [S1] (Fig. 4*B*), quenching the fluorescence to ∼23% of the control (no S1). When saturating MgATP binds to myosin's active site, it weakens the interaction of myosin S1 with actin, which results in restoring pyrene fluorescence (Fig. 4, red).

Vanadate is a phosphate analog that, together with ADP (when vanadate is added during ATP cycling), traps myosin heads in a weak-binding state similar to the M^∗^^∗^.ADP.P_i_ state (34, 35). The addition of saturating vanadate (VO_4_) to actin.S1, under conditions used in the present study, dissociates most of myosin from actin (35). The formation of the ADP.VO_4_-S1 complex relieved the S1-induced inhibition of pyrene fluorescence, increasing the value to 85 ± 0.3% of the myosin-free control (Fig. 4*B*, ). Similarly, the addition of 10 mM potassium pyrophosphate (PP_i_) to the PFA-S1 almost completely restored pyrene fluorescence (Fig. 4*B*, ). Pyrophosphate is known to dissociate the rigor actin–myosin complex almost as effectively as MgATP (36, 37). Thus PFA displays high fluorescence in the absence of strong-binding myosin.

### Steady-state: controls with myosin-specific binding compounds: OM and Mava

**Figure 5:**
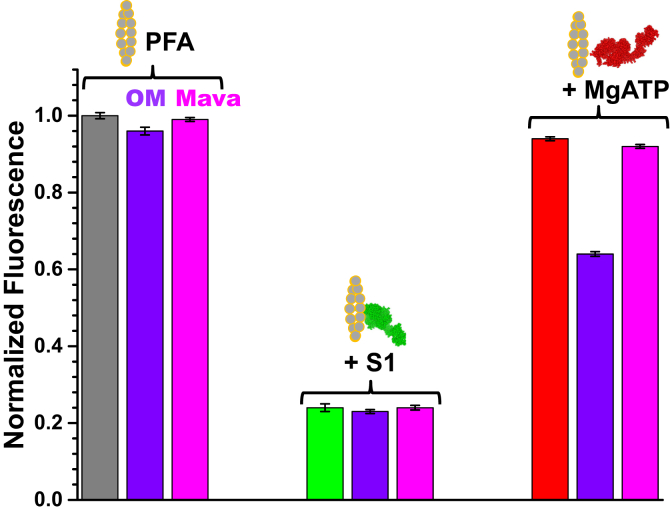
**Effects of myosin-binding drugs on pyrene-labeled F-actin (PFA) fluorescence.** About 5 μM of omecamtiv mecarbil (OM) (*purple*) and mavacamten (Mava) (*pink*) on the fluorescence of 2 μM PFA (*left*, *gray*) and 2 μM PFA.S1 in the absence (*green*, *middle*) and presence (*red*, *right*) of 1 mM MgATP.

OM and Mava, which bind specifically to myosin (16), were used as controls to demonstrate that changes in pyrene fluorescence are due to changes in actin–myosin interaction. The addition of 5 μM OM or Mava did not induce significant changes in the PFA fluorescence, (4% and 1%), respectively (Fig. 5, left, purple and pink bars). When S1 was added (equimolar to actin), the expected decrease in pyrene fluorescence was observed (Fig. 5, middle, green bar), with no effect of OM or Mava (Fig. 5, middle, purple and pink bars). Addition of MgATP restored PFA fluorescence, to 95% in the absence of compound and to 65% in the presence of OM and to 97% in the presence of Mava (Fig. 5), suggesting that OM partially stabilizes the strong-binding state of myosin and actin, while Mava did not. These results are consistent with previous studies showing that OM stabilizes the strong-binding state of myosin to actin during force generation (2, 14, 18) and Mava stabilizes the SRX state, in which myosin is associated with the myosin filament and not the actin filament (6, 20, 21).

### Steady-state: effects of compounds on PFA fluorescence

**Figure 6:**
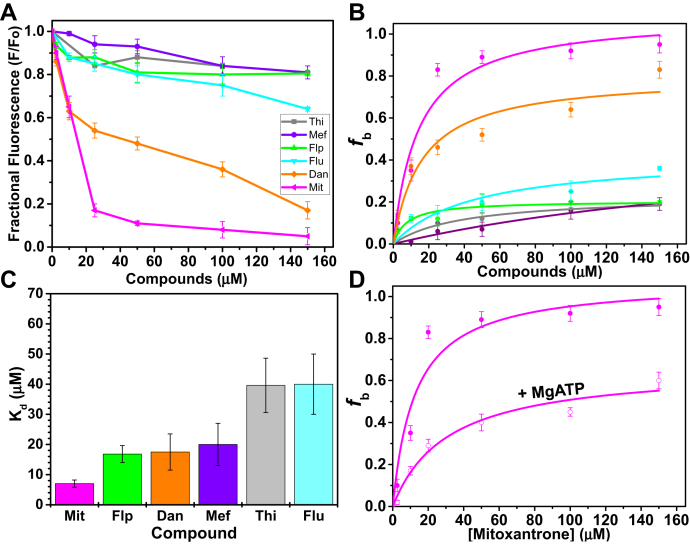
**Effects of compounds on the fluorescence of PFA (2 μM).***A*, dose response of compounds on PFA fluorescence. *B*, the hyperbolic fits of the fraction of actin with bound compound (*f*_b_, Equation 1) *versus* [compound] for some of the compounds. *C*, the dissociation equilibrium constant, *K*_d_ determined from Hill's plots (Equation 3). *D*, *f*_b_*versus* [Mit] showing the hyperbolic dependence on Mit in the absence () and presence () of 200–500 μM MgATP.

Control experiments with the compounds (100 μM) showed minimal emission at 407 nm (0–1.6%), suggesting that the compounds do not contribute to PFA fluorescence emission at 407 nm. The dose–response curves of the compounds in the range of 2.5–150 μM compound show that Car, Teg, Phe, and Hon have minimal effects on the pyrene fluorescence, therefore the *K*_d_ was not determined for these compounds. However, Mef, Flp, Thi, Flu, Dan, and Mit decreased PFA fluorescence with hyperbolic dependence (Fig. 6, *A* and *B*), so the apparent dissociation equilibrium constant, *K*_d_, was determined from fits to the Hill equation (Experimental procedures, Equation 3), as shown for six compounds (Fig. 6, *A* and *C*). Mit, Flp, Dan, and Mef had the smallest *K*_d_ (7.2 ± 1.2 μM, 16.8 ± 2.8 μM, 17.5 ± 6 μM, 20 ± 10 μM, respectively), indicating strong binding to actin. Thi and Flu had *K*_d_ values of 40 ± 10 μM and 39.6 ± 22 μM respectively. The addition of 200-500 μM MgATP had minimal effects on the fluorescence of PFA–compound complexes, except for in the presence of Flp, Mef, and Mit, which showed increased PFA fluorescence in the presence of MgATP. Higher [MgATP] (up to 500 μM) was required to partially restore PFA fluorescence in high [Mit] (100–150 μM), where the *K*_d_ increased from 7.2 ± 1.2 μM to 24.7 ± 5.8 μM, suggesting weaker binding of Mit in the presence of excess ATP (Fig. 6*D*). Similarly, [MgATP] also increased the *K*_d_ of Flp to 25.8 ± 4.2 μM and Mef to 89 ± 28.4 μM.

**Figure 7:**
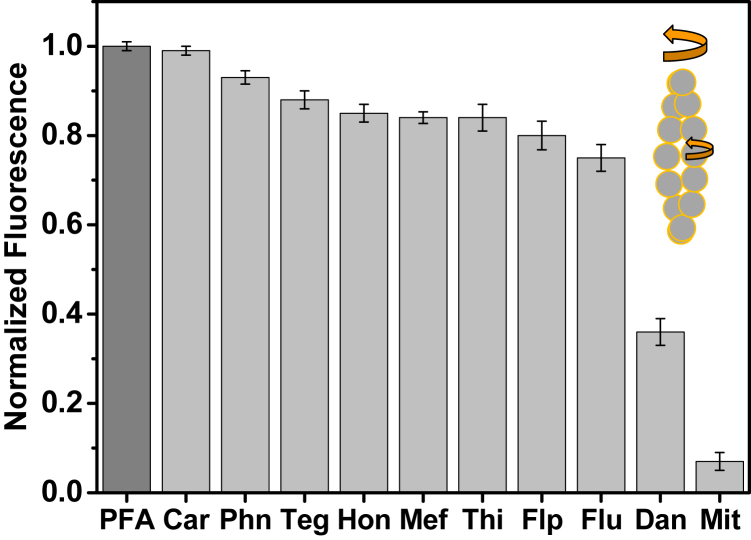
**The effects of compounds on pyrene-labeled F-actin (PFA) fluorescence.** Normalized fluorescence of 2 μM PFA in the absence (*dark gray*) and presence (*gray*) of 100 μM compound. *Inset*, possible conformational changes in actin because of the compound.

The addition of 100 μM compounds to 2 μM PFA decreased the steady-state pyrene fluorescence to varying extents (Fig. 7), indicating that these compounds affect the structure and/or motion of actin (Fig. 7, inset) in the C-terminal region, as previously observed with other optical probes attached to C374 (24). The following experiments were conducted using [compound] that was at least twice the *K*_d_ value: 100 μM compound except for Mit (10 μM), Flu, and Dan (50 μM).

### Steady-state: effects of the compounds on S1 binding and dissociation from F-Actin

**Figure 8:**
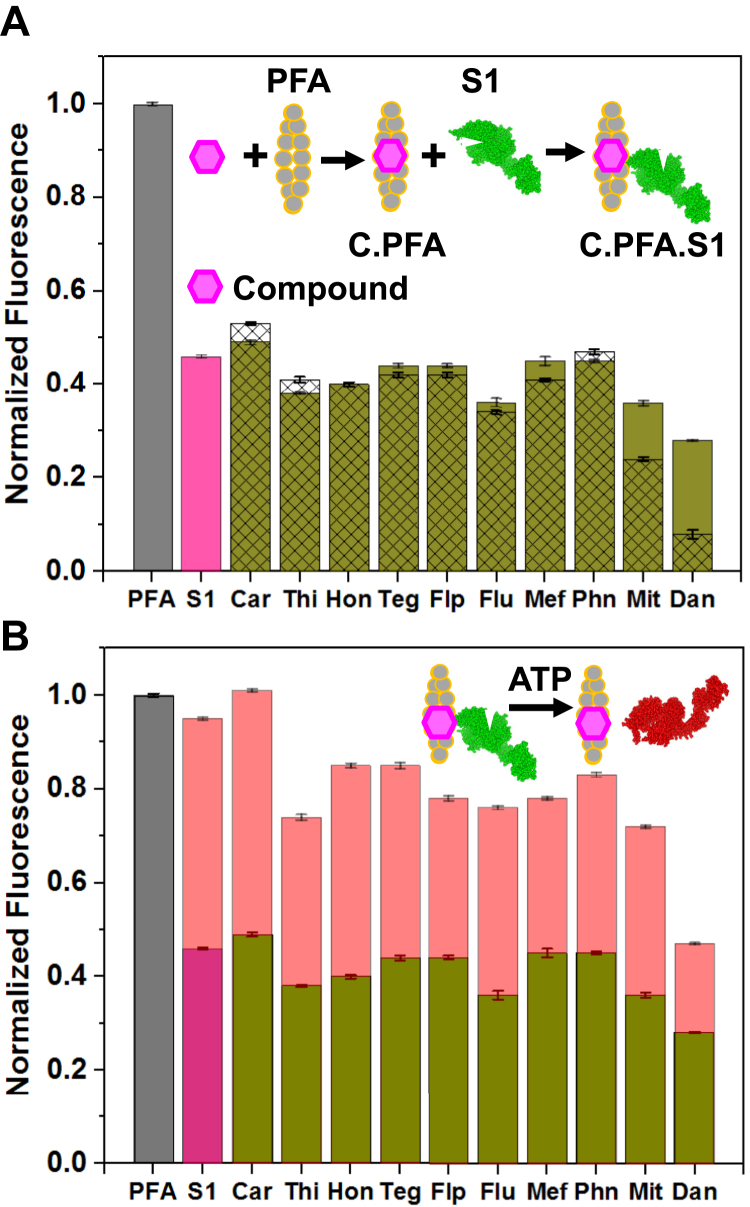
**The effects of S1 binding to the actin–compound complex in the presence and absence of MgATP.** Normalized fluorescence of 2 μM pyrene-labeled F-actin (PFA) (*gray*) with S1 (0.7 S1/actin) only (*pink*). *A,* calculated (using Equation 5, *hatched bars*) and experimental (*green*) fluorescence of PFA.compound.S1 complex. *B,* the effect of 0.5 mM MgATP (*peach*) on the PFA.compound.S1 complex (*green*). [Compound] was 100 μM except for Flu and Dan (50 μM) and Mit (10 μM).

To determine the effect of each compound (C) on S1 binding and ATP-induced dissociation from actin, the strategy was to first create the PFA.C complex and measure PFA fluorescence (Fig. 8, gray bar), then create the PFA.C.S1 complex by adding a substoichiometric ratio amount (0.7:1) of cardiac S1, and measure PFA fluorescence again (shown in Fig. 8*A*, pink bar). We then added Mg.ATP and measured PFA fluorescence (Fig. 8*B*, peach bars). As shown in Figure 4*B* (green circle), the addition of cardiac S1, at a molar ratio of 0.7:1 with actin, induced a 46 ± 0.1% decrease in pyrene fluorescence intensity (Fig. 8, “S1”). Comparison of the calculated PFA fluorescence (*F*_calc_, Equation 5) (Fig. 8*A*, hatched bars) and experimental values (Fig. 8*A*, green bars) of the compound and S1 shows that some of the compounds (Teg, Flp, Flu, Mef, Mit, and Dan) induced a smaller decrease in PFA fluorescence than expected, suggesting a decrease of the fraction of strong-binding myosin. Other compounds (Car, Thi, and Phe) induced a greater decrease in PFA fluorescence, suggesting stronger binding of S1 to actin in the presence of these compounds, and Hon had no effect.

The addition of 500 μM MgATP to the PFA-S1 rigor complex (Fig. 8*B*, peach bars), in the absence of the compound, dissociated the bound S1 and induced nearly full recovery of the PFA fluorescence (Fig. 8*B*, pink bar). However, in the presence of all the compounds except Car, the addition of MgATP to the PFA.C.S1 rigor complex prevented full restoration of PFA fluorescence, indicating that there was strongly bound actin.S1 complex in the presence of the compound (Fig. 8*B*, peach bars).

### Transient-state kinetics: rigor binding of actin–compound complex to S1

**Figure 9:**
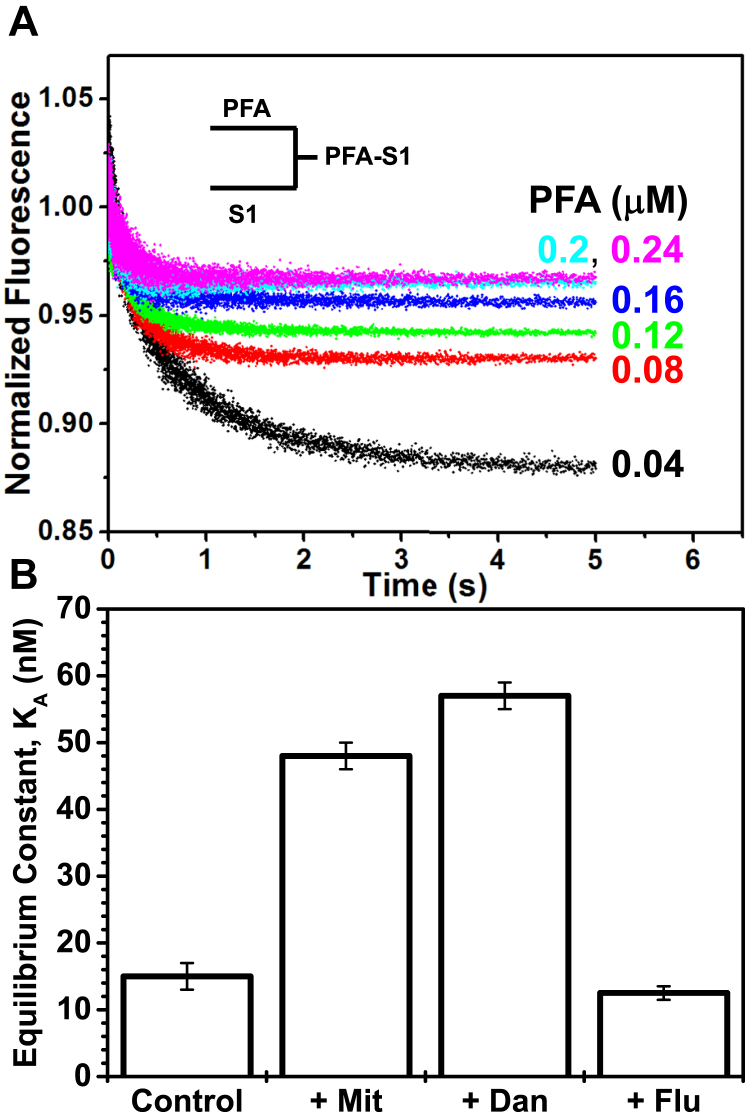
**The effects of rigor binding of S1 to the actin–compound complex.***A,* normalized fluorescence transients of varying [PFA] mixed with 0.02 μM S1 in the absence of compounds. *B*, effects of compounds on dissociation equilibrium constant *K*_A_ for PFA binding to S1. The molar ratio of compound to PFA was 5 for Mit and 25 for Flu and Dan.

The rate constant for binding of actin and S1, *k*_+A_, was determined by varying the PFA concentration (0.04–0.24 μM) in the presence of S1 (0.02 μM), in the absence (control) and presence of the compound (used in the same molar ratio to actin as in the steady-state experiments; PFA: compound for Mit was 1:5, and for Dan and Flu 1:25). The fluorescence of the PFA in the presence of S1 decreased as a function of time and was fitted to a single exponential (Fig. 9*A*). The experiments were done in the presence of apyrase, which removed the fast component that may be due to any residual ATP (see Experimental procedures: Rigor binding of S1 to actin) (28, 38). The rate constant, *k*_obs_, was linearly dependent on [actin], and the slope of the plot of *k*_obs_ *versus* [actin] yielded the second-order rate constant *k*_+A_, while the intercept gave the rate constant for dissociation, *k*_−A_. The dissociation equilibrium constant, *K*_A_, was determined from *k*_−A_/*k*_+A_.

Despite the addition of apyrase to remove any contaminating nucleotide, Dan and Mit induced second component with a slower rate, *k*_obs, slow_, that was insensitive to the [actin]. For PFA binding to β-S1, the dissociation equilibrium constant, *K*_A_, was 15 ± 1 nM, which increased in the presence of Mit and Dan, to 48 ± 2 nM and 57 ± 2 nM respectively, suggesting that these compounds decreased the affinity of rigor actin binding to S1 more significantly than Flu, which induced a dissociation equilibrium constant of 13 ± 1 nM (Fig. 9*B*). This is consistent with the above steady-state results under Effects of the compounds on S1 binding and dissociation from F-Actin that showed a decrease of the fraction of strong-binding myosin in the presence of Mit and Dan. *K*_A_ was not determined in the presence of the other compounds.

The rate of dissociation of S1 from the rigor actin-S1 complex was also determined by the increase of pyrene fluorescence in the presence of three compounds (Mit, Dan, and Flu) in a chase experiment where 20-fold unlabeled F-actin was rapidly mixed with the PFA.C.S1complex (0.25 μM PFA.S1) and monitored for 20 min. The transient curve was best fitted to a double exponential equation to yield a fast and slow rate constant of 0.01 ± 5E-4 s^−1^ and 0.003 ± 4E-4 s^−1^, respectively. No significant change in the rate of dissociation of S1 in the presence of higher [actin] was observed for any of the compounds compared with control PFA-S1 in the absence of compound.

### Transient-state kinetics: ATP binding and isomerization step

**Figure 10:**
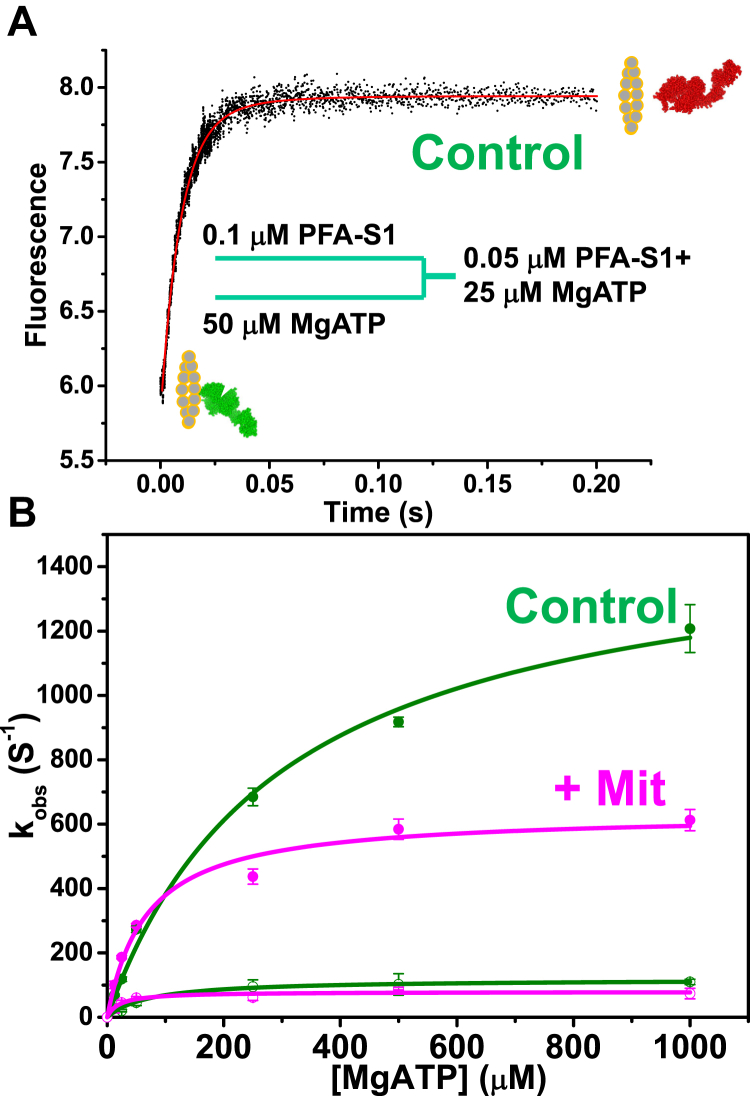
**ATP-induced dissociation of the actin–S1 complex.***A,* transient kinetics of dissociation of the complex of 0.05 μM PFA +0.05 μM cardiac myosin S1 by 25 μM MgATP. *B*, k_obs_*versus* [MgATP] in the absence (*green*) and presence (*pink*) of 0.25 μM Mit. Fast and slow rate constants are *k*_obs1_ (*filled circles*) and *k*_obs2_ (*open circles*). Nonlinear fit to a hyperbolic equation yields *k*_+2_ (Table 1).

To determine the equilibrium constant *K*_1_ for the formation of the collision complex, A.M.T, the rigor complex of PFA and myosin-S1 complex was formed in one syringe and the Mg.ATP placed in another syringe. Rapid mixing results in a rapid increase of pyrene fluorescence as ATP dissociates the S1 (Fig. 10*A*). This biphasic transient curve is best fitted to a double exponential equation to yield a fast rate constant *k*_obs1_ and slow *k*_obs2_, with corresponding large amplitude, *A*_1_ and small amplitude *A*_2_. For the transient curve in Figure 10*A*, 50 nM PFA and S1 (1:1) in 25 μM MgATP (final concentrations) has a fast phase and large amplitude (118 ± 7 s^−1^, 80%) and slow phase with a small amplitude (20 ± 3.5 s^−1^, 20%). The *k*_obs2_ (*k*_+α_) and the amplitude *A*_2_ did not show significant dependence on the varying [ATP]. The slow phase had a *k*_obs2_ of 63 ± 13 s^−1^ (Table 1). The *k*_obs1_ showed hyperbolic dependence on [ATP] with myosin S1 in the absence (control) (Fig. 10*B*, green, filled circles) and in the presence of Mit (Fig. 10*B*, pink, filled circles), but *k*_ob2_ is relatively independent of [MgATP] for control and Mit and saturates at 118 ± 79 s^−1^ and 79 ± 20 s^−1^, respectively (Fig. 10*B*, open circles). The *k*_obs1_ for the other nine compounds showed similar hyperbolic dependence on [MgATP] that ranged between the control and that observed for Mit (Fig. 10*B*). At low concentrations of MgATP, the observed rate constant *k*_obs1_ shows linear dependence, typical of a pseudo second-order reaction, and describes the second-order rate constant for nucleotide binding to the actin–myosin complex, *K*_1_*k*_+2_, which is obtained from the slope of the linear plot of k_obs1_ *versus* low [MgATP] (Equation 10). The rate constant *k*_+2_ was determined from hyperbolic plots of *k*_obs1_ *versus* [MgATP] (Equation 11) for ATP binding to the rigor actin-S1 complex (Fig. 2). The association equilibrium constant *K*_1_ was determined from the ratio of *K*_1_*k*_+2_/*k*_+2_.

**Table 1:** Kinetic parameters (defined in Fig. 2) for the effects of compounds on ATP binding and isomerization in the actin–compound-S1 complex

Experimental conditions for stopped-flow experiments: F-Mg buffer, 25 °C. Values are mean ± SEM for four to seven replicates. ORIGIN fitting errors are reported for calculated equilibrium and rate constants. The effects for 1/*K*_1_ and *k*_+2_ are categorized as mild (*blue*): <1.5-fold; moderate (*violet*): 1.5- to 3-fold; and severe (*pink*): >3-fold.

For the control sample of actin and S1, we determined *K*_1_*k*_+2_, 1/*K*_1_, and *k*_+2_ values of 3.83 ± 0.25 μM^−1^s^−1^, 421 ± 34 μM, and 1621 ± 78 s^−1^ (Table 1), respectively, which are consistent with that observed previously for cardiac β-S1 where *K*_1_*k*_+_2, 1/*K*_1_, and *k*_+2_ were 4.4 ± 0.3 μM^−1^s^−1^, 328 ± 53 μM, and 1543 ± 100 s^−1^ (8). All the compounds except Car and Hon induced modest increases in the range of 1.34–1.76-fold in the second-order rate constant *K*_1_*k*_+2_ for ATP binding (Table 1).

**Figure 11:**
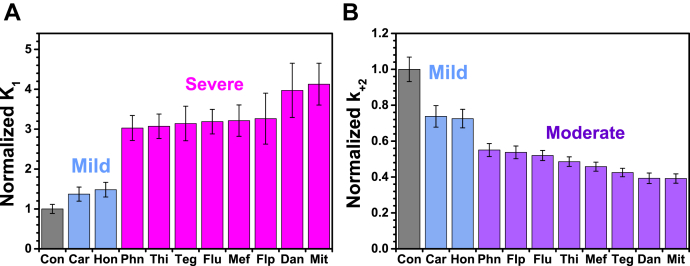
**The effects of compounds on MgATP binding and the subsequent isomerization step in the actin–S1 complex.***A,* normalized equilibrium constant *K*_1_ (Fig. 2) for ATP binding to S1 in the rigor PFA.S1 complex (0.05 μM) in the absence (Con) and presence of each compound. *B*, normalized rate constant *k*_+2_, for the isomerization to the weak-binding state. Before normalization, the Con values are *K*_*1*_ = 2.4 ± 0.03 nM^−1^ and *k*_*+*2_ = 1620 ± 80 s^−1^ (Table 1). The effects for *K*_1_ and *k*_+2_ are categorized as mild (*blue*, <1.5-fold), moderate (*violet*, 2–3-fold), or severe (*pink*, >3-fold). [Compound] was 100 μM, except Mit was 5-fold and Flu and Dan were 25-fold over [PFA].

All of the compounds induced decreases to varying extents in 1/*K*_1_ and *k*_+2_ (Table 1, Fig. 11, *A* and *B*). Compared with the control values, we defined mild effects to be <1.5-fold, moderate effects to be within 1.5–3-fold, and severe effects to be >3-fold. Thus effects of Car and Hon on *K*_1_ are mild (Fig. 11*A*; shown as 1/*K*_1_ in Table 1, blue), while those of the other compounds are severe (Fig. 11*A*, shown as 1/*K*_1_ in Table 1, pink). The increased *K*_1_ values indicate increased affinity of ATP for actin-bound myosin.

The rate constant, *k*_+2_, for the isomerization of the strong-binding collision complex (A.M.T) to the weak-binding ternary complex (A∼M.T) was determined from hyperbolic fits of *k*_obs1_ *versus* [MgATP] (Equation 10) in (shown in Fig. 11). Dan and Mit showed the largest decrease (>2.5-fold) (Fig. 11*B*, violet; Table 1) compared with the control sample (Fig. 11*B*, gray). Flp, Phn, and Flu also induced moderate decreases (1.8–1.9 fold) (Fig. 11*A*, violet). Thi, Mef, and Teg also induced moderate decreases (2–2.4 fold) (Fig. 11*A*, violet), while Car and Hon did not induce significant changes (<1.5 fold) (Fig. 11*B*, blue; Table 1).

We categorize the effect of the compound as severe if at least one parameter (*K*_1_ or *k*_+2_) was affected >3-fold. Therefore, Mit, Dan, Phn, Thi, Teg, Flu, Mef, and Flp were determined to induce severe effects to varying degrees, and Car and Hon induced mild effects.

The compounds did not significantly affect the average rate constant of the slow component, *k*_obs2_ (*k*_+α_) or the ratio of the amplitudes *A*_1_ and *A*_2_ (the equilibrium constant, *K*_α_), for the isomerization of actin-bound myosin from the closed (A.M) to open (A.M′) configuration (Fig. 2) to allow ATP binding to myosin (Table 1).

In terms of the extent of compound effects, our steady-state kinetics results (Fig. 8) are consistent with the transient-state results (Fig. 11). Both studies show that Car and Hon induced mild effects. Phn, Flp, Flu, Thi, Mef, and Teg induced severe effects to varying degrees, while Dan and Mit induced the most severe effects on rigor actin-S1 binding and release with MgATP.

## Discussion

### Pyrene-actin fluorescence

The steady-state results show that the binding of cardiac S1 to F-actin linearly decreased the pyrene fluorescence up to a molar ratio of 1:1, as we previously showed (20). The inhibition of fluorescence is relieved by MgATP and nucleotide analogs that induce the weak binding or dissociated myosin (Fig. 4).

The addition of the compounds to PFA decreased the pyrene fluorescence to varying extents (Fig. 6). The dissociation equilibrium constant *K*_d_ was determined from the change in PFA fluorescence as a function of [compound]. Mit, Flp, Dan, Thi, Mef, and Flu had a *K*_d_ that ranged from ∼ 5 to 50 μM, compared with the compounds that likely had a *K*_d_ > 100 μM. The compounds that showed tighter binding to actin (*K*_d_ < 50 μM) (Fig. 6) are the compounds that induced the largest perturbation in the actin–myosin interaction (Fig. 11, Table 1). Our observation that the compounds directly affect the pyrene fluorescence of F-actin suggests that the binding of the compounds to actin induces a conformational change that exposes the pyrene moiety to the solvent, rendering it susceptible to collisions with other molecules that decrease its fluorescence. Our observation that MgATP relieves the fluorescence inhibition for some compounds (Mit, Flu, and Mef) suggests that those compounds probably bind near the nucleotide pocket in F-actin, which induces a conformational change that perturbs the bound compound, therefore, relieving the conformational change imposed at C374 in actin. In fact, Mit was identified in another HTS screen, which showed that Mit prevents GTPase binding to RhoGTApases (RhoA/RacA/Cdc42) and that Mit binds close to the GTP active site (39). It is unknown whether the compounds completely dissociate from F-actin in the presence of MgATP.

### Rigor binding of actin and S1

Some of the compounds inhibited the S1-induced decrease of PFA fluorescence in the absence of ATP (rigor) (Fig. 8*A*), suggesting decreased affinity of S1 for actin. We determined the rigor binding of actin to S1 by varying [actin] in the range of 2–12-fold in excess of S1 during the stopped-flow fluorescence transient kinetic experiments. The equilibrium dissociation constant, *K*_A_, for S1 was 15 ± 2 nM (Fig. 11). This is consistent with previous reports of cardiac S1 from various species; for tissue-purified bovine cardiac β-S1 *K*_A_ was 6.3 nM (40), for tissue-purified human cardiac (HC) S1 *K*_A_ was 8 ± 2 nM or 10 ± 1.8 nM (8), and for bovine masseter β-S1 *K*_A_ was 7 nM (41). The dissociation equilibrium constant, *K*_A,_ for actin binding to S1 in the presence of Mit and Dan, was 3-fold and 3.8-fold higher, respectively (Fig. 11), suggesting that these compounds decreased the binding or association of the PFA.compound complex with S1, which is consistent with the fluorescence results (Fig. 8*B*) that showed incomplete S1 decrease of pyrene fluorescence in the presence of the compounds, implying a smaller fraction of actin containing strongly bound myosin. Flu induced a smaller inhibition of the S1-induced decrease in the pyrene fluorescence, and the measured *K*_A_ was near normal values.

### ATP binding and actin-S1 isomerization

All of the compounds except Car prevented the full restoration of PFA fluorescence (Fig. 8*B*) by ATP-induced dissociation of the actin-S1 complex, indicating that the compounds induce conformational changes in actin that affect the myosin ATPase cycle. The steady-state and transient-state results are consistent with each other in that they both show an increase in the population of actin that is strongly bound to myosin in the presence of saturating ATP. This was confirmed by transient-state kinetics measurements showing that several of the compounds increased the second-order rate constant *K*_1_*k*_+2_, increased the affinity *K*_1_ of ATP for actin-bound S1, and decreased the subsequent rate constant *k*_+2_ for the isomerization of the collision complex (A.M.T) to the ternary complex (A∼M.T) (Figs. 2 and 11). Therefore, we conclude that several of the compounds shift the equilibrium (*K*_1_ in Fig. 2) toward stronger ATP binding in the myosin active site and subsequently decrease the rate (*k*_+2_) of the strong-to-weak transition that actin–myosin interaction undergoes early in the ATPase cycle. This is in contrast to control myosin where the tighter a nucleotide binds to S1, the more the equilibrium moves toward weaker actin binding (42).

We categorized the compounds into three groups based on the extent of the effects: mild (Car and Hon) and severe to varying degrees (Flp, Thi, Teg, Mef, Phn, Flu, Mit, and Dan).

### Interpretation and relevance of results

Although the second-order rate constant, *K*_1_*k*_+2_, changed by a factor of less than 2 for these compounds, in several cases *K*_1_ and/or *k*_+2_ changed by more than a factor of 2, so it is important to consider these rate constants individually. At least eight compounds (Phn, Flp, Flu, Thi, Mef, Teg, Dan, and Mit) increased *K*_1_ and decreased *k*_+2_ by at least a factor of 2 (up to a factor of 4 in some cases), thus increasing the affinity of ATP and slowing the rate of isomerization, respectively. Similar effects have been observed for myosin HCM mutations that showed little or no change in *K*_1_*k*_+2_ or *k*_cat_, but showed significant decreases in *k*_+2_ or 1/*K*_1_ (8). For several of those HCM mutations, these were among the most significant effects, and it was pointed out that changes greater than 20% can be important (8). We observed changes greater than 20% for *K*_1_*k*_+2_, 1/*K*_1_, and *k*_+2_ (Table 1).

The relevance of incremental small changes is important, as discussed by Spudich (6) with respect to HCM and DCM myosin mutations. Spudich's premise is that HCM hypercontractility could be the result of small changes in one or more kinetic steps that lead to “*more myosin heads being in the A.M.ADP state or remaining in the A.M.ADP state longer than normal, resulting in an increase in ensemble force in the muscle*.” While the changes we observe are not in the rate-limiting step for cardiac S1 in solution, changes in kinetic steps preceding (early steps such as ATP binding and isomerization) the rate-limiting step can influence changes in this step. Spudich further acknowledges that despite the deleterious effects of the HCM or DCM phenotypes, the expected changes in A.M.ADP may be small (10–30% for HCM) since “primary contractility changes are relatively small” (6). Similarly, Geeves and coworkers (43) state, *“Note that for these ATPase cycles, the concept of a simple rate-limiting step does not apply, as has been recognized in enzymology for a number of years. Multiple states contribute to the overall balance of the cycle, and small changes in any step can and will alter the balance of events in the cycle.”* This is also applicable when studying the mechanism of action of compounds as potential drug candidates.

### Mechanism of compound inhibition

Because these compounds increase the equilibrium association constant *K*_1_, and decrease the rate constant *k*_+2_ (*k*_max_), their mode of action is uncompetitive inhibition, in which the inhibitor binds to and stabilizes the enzyme–substrate complex allosterically (44). These allosteric effects are likely, as actin is known to act in a cooperative manner to affect either other actin monomers or myosin (45). It was previously shown that the myosin steady-state ATPase activity is unaffected by the presence of the compounds in the absence of actin (24), so inhibition is through conformational changes in actin that affect the M.ATP and M.ADP.P complex while bound to actin. This supports our hypothesis that the actin-binding compounds allosterically affect the actin–myosin interaction by perturbing kinetic constants early in the myosin ATPase cycle.

### Relationship to other work

Previous work in this laboratory showed that FRET from a fluorescent donor on the C terminus of actin to an acceptor on the N-terminal extension of the cardiac ventricular myosin ELC is sensitive to disease-causing mutations in myosin ELC (33). A subsequent study showed that a 12-amino-acid peptide derived from the ELC N-terminal extension, also labeled with an acceptor, is sufficient to detect this interaction for the purposes of HTS (24, 25). This led to the discovery of the ten actin-binding compounds studied in this paper, each of which causes a decrease in FRET efficiency, indicating that these compounds weaken the binding of cardiac myosin-derived peptide to actin (24). This was confirmed by the observation that the steady-state actin-activated myosin ATPase activity was decreased by several of the compounds (24). In a subsequent study, they showed that several of these compounds induce significantly different effects on the Ca^2+^-dependence of steady-state ATPase activities of cardiac and skeletal myofibrils (27). Our steady- and transient-state results are essentially consistent with these perturbations in the steady-state actin-activated myosin ATPase activities.

Some of the compounds (Flp, Car, Mit, and Thi) reduced the polymerization rate (increased the half time (t_1/2_)) of skeletal and cardiac actin, but only Phn, Teg, and Thi showed isoform-specific differences, while Hon, Dan, and Mef did not have significant effects (27). Also, the compounds did not affect the depolymerization rate of either skeletal or cardiac actin (27).

### Structural mechanism of action of the compounds

Holmes and co-workers (46) showed that the lower 50kd domain of myosin initially binds in a weak stereospecific interaction with actin (46) and that during this process the myosin nucleotide cleft is closed. On subsequent strong binding of myosin, the upper 50kd domain seems to swing round so that the cardiomyopathy loop comes into contact with the actin surface (46). The switch 1 element of the nucleotide-binding site is anchored in the upper 50kd domain, so that the strong binding to actin results in a 6–9 Å movement of switch 1 (46) that appears to open the nucleotide-binding pocket to allow binding of the nucleotide, which then closes and induces the 50kd cleft to open, thus weakening the actin–myosin interaction to dissociate the complex (4) (Fig. 2).

Our steady-state results show that some of the compounds (Mit, Dan, Mef, Flu, Flp, and Teg) decrease the effect of S1 on PFA fluorescence, suggesting weaker actin binding, while other compounds (Phn, Thi, and Car) increase the effect of S1, suggesting stronger actin binding.

The compounds that decreased strong binding of S1 to actin also significantly increased the equilibrium constant *K*_1_ for ATP binding to actin-S1. These compounds also decreased the subsequent rate of isomerization from the collision complex (A.M.T) to the ternary complex (A∼M.T), thus slowing the S→W transition that myosin undergoes early in the ATPase cycle. Therefore, it is likely that structural changes induced by these compounds in actin allosterically affect the myosin kinetics.

### Potential therapeutic applications

Two compounds that we categorized as severe (Mit, Teg) have been shown to induce heart failure. Thi and Flp (moderate-to-severe effects in the present study) were removed from clinical use because they caused liver toxicity, and Dan (severe effect in this study) is under FDA caution because it causes hepatoxicity. The other five compounds induce either mild (Car and Hon) or varying severe (Flu, Phn, and Mef) effects. Although most of the compounds in the present study have been used to treat noncontractile dysfunction in humans (as anticancer, antipsychotic, and antimalarial agents), it is possible that some of them, or derivatives of them, have therapeutic potential for cardiomyopathy where attenuation of contractility is beneficial, as in a hypercontractile state caused by HCM mutations in myosin or actin (6, 47). Regulation of striated muscle contractility is not only thin-filament-dependent, but also thick-filament-dependent, whereby the myosin-binding affinity to actin is attenuated *via* phosphorylation of the light chains (48) and myosin-binding protein C (49, 50), changes in flexibility and conformation (LC domain (51), converter region (52), actin-binding region (7, 53)), and small molecules (Mava and OM) (47) that affect the myosin ATPase cycle. In the present study, we have shown that the compounds increase ATP affinity and decrease the rate constant for the subsequent isomerization step in ternary complex formation (and hence the release of S1 from actin), which is likely to produce actin-bound nonforce-producing heads, resulting in decreased myosin ATPase activity. It is possible that some of these actin-binding compounds will prove to be useful in decreasing hypercontractility in the HCM disease, caused by either actin or myosin mutations, by attenuating the binding affinities of myosin for actin.

## Conclusions

Starting from compounds previously identified from high-throughput screening, we have shown that pyrene-labeled actin is sensitive to conformational changes that these compounds induce in F-actin and that these conformational changes affect the strong-binding state of myosin to actin. Some of these compounds prevent cardiac myosin S1 from decreasing pyrene-actin fluorescence in the absence of ATP, implying that they decreased the fraction of strongly bound myosin in rigor. Many of the compounds prevent full restoration of fluorescence from the actin-S1 complex in ATP, implying that they decrease the fraction of nucleotide-bound myosin or that the actin-attached states are more populated in the presence of ATP.

Transient kinetics shows that the affinity for actin binding to S1 in rigor is weaker (increased equilibrium constant, *K*_A_) in the presence of Mit and Dan. In addition, the binding affinity of ATP for myosin in the rigor actin–myosin complex is increased for most of the compounds (possibly enhancing the formation of the collision complex, A.M.T), which then induces a subsequent decrease in the rate constant for the isomerization of the collision complex to the ternary complex, A∼M.T. Most of the other compounds show a large increase in *K*_1_, but a smaller decrease in *k*_+2,_ thus revealing enhanced affinity for ATP in the actin–myosin complex and decreased rate of the subsequent isomerization step that results in impaired actin-activated myosin ATPase activity. Therefore, the present study shows that the early steps of the cardiac myosin ATPase kinetics cycle are affected by myosin structural changes induced by compounds binding to actin. To gain further insight into the mechanisms of action of these compounds, studies are needed to resolve the kinetics of the later steps (*e.g.*, ATP hydrolysis and product release). Most importantly, this research also sets the stage for studying the mechanism of action of actin-binding compounds discovered in future HTS studies.

## Experimental procedures

### Preparations and assays

Acetone powder was prepared from young female New Zealand White rabbit skeletal muscle (54). Rabbit skeletal F-actin was prepared as described previously from acetone powder (55). Bovine cardiac β-myosin and α-chymotryptic S1 were prepared from bovine left ventricular muscle (Pelfreez Biologicals) (2, 56) and stored in sterile 150 mM sucrose at −80 °C until needed. The actin-activated myosin S1 steady-state MgATPase activity was measured using the NADH-coupled assay at 25 °C using 15 μM phalloidin-stabilized F-actin and 0.1 μM cardiac β-S1 in F-Mg buffer (10 mM Tris (pH 7.5), 3 mM MgCl_2_) (20, 33). ATPase activity was reported as the mean ± SEM s^−1^ (n = 4).

The ten compounds were obtained from Target Molecule Corp and stored at –20 °C as 10 mM stock solutions in DMSO until use. OM and Mava were obtained from Selleck Chemicals and stored as 10 mM stock solutions at –20 °C until use. Sodium vanadate (NaVO_4_) and potassium pyrophosphate (PP_i_) were obtained from Sigma-Aldrich, and stock solutions of 50 mM and 1 M, respectively, were prepared as described previously (36). Since vanadate does not bind significantly to actin-S1 (A.M) complex (57), to form the vanadate complex with myosin S1, first 0.3 mM MgATP was added to an equimolar PFA.S1 (2 μM) complex and allowed to hydrolyze, then 0.3 mM NaVO_4_ was added to the complex (58). To study the effect of pyrophosphate, 10 mM PPi was added to an equimolar PFA.S1 (2 μM) complex. These ligand concentrations were saturating.

### Labeling F-Actin

F-actin was labeled with N-(1-pyrene) iodoacetamide (PIA) (Invitrogen, Thermo Fisher Scientific) essentially as described previously (28, 31). Briefly, F-actin (1 mg/ml) in F-Mg buffer + 0.2 mM MgATP was mixed with PIA at a molar ratio of 1:10 and left overnight at room temperature in the dark. Then, 10 mM DTT was used to stop the reaction, and the labeled F-actin was sedimented at 80K for 45 min at 4 °C. The pellet was resuspended with a dounce homogenizer in G-buffer (5 mM Tris [pH 7.5], 0.5 mM ATP, 0.2 mM MgCl_2_), centrifuged for 10 min at 70K to remove the PIA precipitate. The supernatant was repolymerized with 3 mM MgCl_2_, and the labeled F-actin was collected in the pellet after centrifugation at 80K for 45 min, then resuspended with a dounce homogenizer with F-Mg buffer. The pyrene concentration on F-actin was determined by absorbance (344 nm) and extinction coefficient of 2.2 × 10^4^ M^−1^ cm^−1^. The labeled actin concentration was determined by Bradford assay (Biorad). A typical dye:protein ratio of 0.9 to 0.95 was obtained for pyrene-labeled actin. The PFA was stabilized with phalloidin (Sigma-Aldrich) at a 1:1 M ratio, stored on ice, and used within a week.

### Fluorescence spectroscopy

Fluorescence spectra of 2 μM PFA in F-Mg buffer were acquired with a Cary spectrophotometer. The sample was placed in a cuvette in a temperature-controlled holder and excited with a Xenon flash lamp at 365 nm with 5 nm slit width. Emission fluorescence intensity was acquired and averaged for 30 s at 407 nm with a 10 nm slit width. A final concentration of 2 μM PFA in F-actin buffer was used. Fluorescence intensity (*F*) was corrected for each compound's emission at 407 nm. Control experiments with the compounds in the absence of PFA showed minimal fluorescence emission (<1%) at 407 nm. Control experiments with 1–1.5% DMSO showed negligible effect on PFA fluorescence.

The binding of a compound to PFA was determined from the dose-dependent change of fractional fluorescence *f*_f_ (*F*/*F*_0_), where *F* and *F*_0_ are PFA fluorescence in the presence and absence of compound, respectively, in the range of 0–150 μM compound concentration [C]. The fraction of actin bound to the compound (*f*_b_, Equation 1) was determined for the compounds that showed hyperbolic dependence (Equation 2) of PFA fluorescence (*F*_0_) as a function of [C]. The dissociation equilibrium constant (*K*_d_) for the compound binding to PFA was determined from nonlinear fits to Hill's equation (Equation 3) for plots of *f*_f_ *versus* [C] using the program ORIGIN (OriginLab Corp., MA), where *f*_Start_ and *f*_*End*_ are the maximum and minimum values of *f*_f_, and *n* is the Hill's coefficient of cooperativity (59).

(1) $$f_{b}=1-\left(F/F_{0}\right)$$

(2) $$f_{b}=f_{\max}\left[C\right]/\left(K_{d}+\left[C\right]\right)$$

(3) $$f_{f}=f_{\left(Start\right)}+\left(f_{End}-f_{Start}\right)C^{n}/\left(K_{d^{n}}+C^{n}\right)$$

The fractional saturation of pyrene actin fluorescence due to S1 binding to actin (*f*_b_) was determined by normalization relative to the PFA fluorescence, as described in Equation 4 (38):

(4) $$f_{b}=\left(F_{0}-F\right)/\left(F_{0}-F_{\infty }\right)$$

*F*_0_ is the PFA fluorescence in the absence of S1, *F*_*∞*_ is the PFA fluorescence at infinite [S1], and *F* is the PFA fluorescence in the presence of S1 and/or compound.

### Rigor binding of S1 to F-actin-compound complex

To determine whether a compound affected the binding of S1 to F-Actin in rigor (the absence of nucleotide), the expected decrease in SS fluorescence intensity (*F*_calc_) was calculated from experimental values of *F*_*Compound*_ and *F*_*0.7:S1*_ (Equation 5). To ensure that a negative fluorescence intensity was not induced by the additive effect of the compound and S1, a ratio of 0.7:1 of S1:actin was used in the experiment. *F*_calc_ was determined from normalized fluorescence intensity:

(5) $$F_{calc}=\left(F_{Compound}+F_{0.7S1}\right)$$

### Transient-state fluorescence spectroscopy

Transient kinetics experiments to detect the total fluorescence intensity were performed using an applied photophysics stopped-flow spectrophotometer that is capable of single-mix and sequential-mix experiments with temperature control (20). Experiments were performed at 25 °C with filtered and degassed solutions. The instrument dead time for single-mix experiments was 1.3 ms. Samples were excited at 365 nm, and pyrene emission fluorescence was acquired with a 2 mm path length and 400 nm long-pass filter. Data analysis was done using the program, ORIGIN (OriginLab Corp).

### Analysis of stopped-flow fluorescence data

Fluorescence decays from the transient kinetics measurements were fitted to single (Equation 6) and double (Equation 7) exponential equations. The best fit was evaluated by the lowest χ^2^ value of the fit compared with the experimental data.

(6) $$\Delta F\left(t\right)=\Delta A_{1}\;\exp\left(-k_{1}t\right)+F_{\infty }$$

(7) $$\Delta F\left(t\right)=\Delta A_{1}\;\exp\left(-k_{1}t\right)+\Delta A_{2}\;\exp\left(-k_{2}t\right)+F_{\infty }$$

Δ*F* is the change in PFA fluorescence, *F*_*∞*_ is the fluorescence at infinite [substrate], *A* and *k* are the observed amplitude and rate constant of the fluorescence change, respectively.

### Rigor binding of S1 to actin

As PFA fluorescence is very sensitive to rigor binding of myosin, we determined the effect of the compound on the binding of S1 to actin. The rate of binding, *k*_+A_ was determined by varying the PFA concentration (0.04–0.24 μM) in the presence of 0.02 μM S1 in F-Mg buffer. To remove effects of ATP or ADP, the mixed actin-S1 samples were treated with three units of apyrase per mL for 5 min at room temperature before fluorescence acquisition. Addition of apyrase removed contaminating nucleotide, resulting in single-exponential decays for the control experiments with cardiac S1 only (28, 38). To determine the effect of the compounds on S1 binding to actin, PFA was mixed with the compound in one syringe and rapidly mixed with S1 from the second syringe to obtain *k*_obs_. The compound:PFA ratios from the steady-state experiments were used: [Mit] was 5-fold and [Flu] and [Dan] were 25-fold over the [PFA]. The linear plot of *k*_obs_ *versus* [PFA] yielded a slope that described the second-order rate constant (*k*_+A_) of S1 binding to actin, while the intercept described the rate constant rate of S1 dissociation (*k*_−A_) (Fig. 1, Equation 8). The equilibrium constant for dissociation, *K*_A_, was determined from the ratio of the rate constants *k*-_A_/*k*_+A_ (Fig. 1, Equation 9).

(8) $$k_{obs}=\;k_{+A}\left[PFA\right]+k_{-A}$$

(9) $$K_{A}=k_{-A}/k_{+A}$$

### MgATP binding and isomerization step

The detachment of S1 from actin involves the binding of ATP (T) to the open configuration of A.M (Fig. 2), which is controlled by the diffusion-limited association equilibrium constant *K*_1_, in the formation of the collision complex (A.M.T), which is followed by the (*k*_+2_) isomerization to A∼M.T, in which actin is weakly bound and ATP is more strongly bound to myosin (Fig. 2) (28). This is rapidly followed by dissociation of myosin S1 from actin at a rate of *k*_dissoc_.

(10) $${k_{obs}}_{,fast}=K_{1}k_{+2}\left[ATP\right]/\left(1+\;K_{1}\left[ATP\right]\right)$$

(11) $$k_{obs,fast}=k_{+2}\left[ATP\right]/\left(K_{0.5}+\left[ATP\right]\right)$$

*K*_1_ was determined by mixing the rigor complex of PFA.S1 (A.M) (final concentration of 0.05 μM) with varying final concentrations of MgATP (12.5–1000 μM) in F-Mg buffer in the absence and presence of the compounds, whereby the PFA.compound complex was first made, then mixed with S1, and placed in one syringe, and MgATP solution was placed in the other syringe. The transient curves were fitted to rate constants, *k*_obs, fast_ and *k*_obs, slow_, and with corresponding amplitudes *A*_1_ and *A*_2_. At low concentrations of ATP (*K*_1_.MgATP << 1), the plot of the observed rate constant (*k*_obs, fast_) *versus* [MgATP] shows a linear dependence that is typical of a pseudo second-order reaction, from which the slope is the apparent second-order rate constant, *K*_1_*k*_+2_ (Equation 10). However, at higher concentrations of MgATP, the relationship becomes hyperbolic (as described in Equation 11) in which a plot of *k*_obs_ *versus* [Mg.ATP] plateaus at *k*_max_, which corresponds to rate constant *k*_+2_. The dissociation equilibrium constant of the actin-S1 complex *K*_0.5_ (= 1/*K*_1_) is determined from *k*_+2_/*K*_1_*k*_+2_ and is the ATP concentration at half saturation that describes the binding affinity of ATP for the actin–myosin complex.

## Data availability

All data described are contained within the article.

## Conflict of interest

D. D. T. holds equity in, and serves as President of, Photonic Pharma LLC. This relationship has been reviewed and managed by the University of Minnesota. Photonic Pharma had no role in this study. The authors declare no conflicts of interest in regard to this article.
